# Genomic signatures for predicting survival and adjuvant chemotherapy benefit in patients with non-small-cell lung cancer

**DOI:** 10.1186/1755-8794-5-30

**Published:** 2012-07-02

**Authors:** Ryan K Van Laar

**Affiliations:** 1 ChipDX LLC, PO Box 286874, New York, NY, 10128, USA

## Abstract

**Background:**

Improved methods are needed for predicting prognosis and the benefit of delivering adjuvant chemotherapy (ACT) in patients with non-small-cell lung cancer (NSCLC).

**Methods:**

A novel prognostic algorithm was identified using genomic profiles from 332 stage I-III adenocarcinomas and independently validated on a separate series of 264 patients with stage I-II tumors, compiled from five previous studies. The prognostic algorithm was used to interrogate genomic data from a series of patients treated with adjuvant chemotherapy. Those genes associated with outcome in the adjuvant treatment setting, independent to prognosis were used to train an algorithm able to classify a patient as either a responder or non-responder to ACT. The performance of this signature was independently validated on a separate series of genomic profiles from patients enrolled in a randomized controlled trial of cisplatin/vinorelbine *vs.* observation alone (JBR.10).

**Results:**

NSCLC patients exhibiting the high-risk, poor-prognosis form of the 160-gene prognosis signature experienced a 2.80-times higher rate of 5-year disease specific death (log rank P < 0.0001) compared to those with the low-risk, good prognosis profile, adjusted for covariates. The prognosis signature was found to especially accurate at identifying early stage patients at risk of disease specific death within 24 months of diagnosis when compared to traditional methods of outcome prediction.

Separately, NSCLC patients with the 37-gene ACT-response signature (n = 70, 64 %), benefited significantly from cisplatin/vinorelbine (adjusted HR: 0.23, P = 0.0032). For those patients predicted to be responders, receiving this form of ACT conferred a 25 % improvement in the probability of 5-year-survival, compared to observation alone and adjusted for covariates. Conversely, in those patients predicted to be non-responders, ACT was observed to offer no significant survival benefit (adjusted HR: 0.55, P = 0.32).

The two gene signatures overlap by one gene only *SPSB3*, which interacts with the oncogene MET. In this study, higher levels of SPSB3 which were associated with favorable prognosis and benefit from ACT.

**Conclusions:**

These complimentary prognostic and predictive gene signatures may assist physicians in their management and treatment of patients with early stage lung cancer.

## Background

Non-small cell lung cancers (NSCLC), including adenocarcinoma, squamous and large-cell tumors, represent 85 % of all lung tumors and result in 1.9 million deaths each year [1]. While disease stage is associated with outcome and commonly used to determine adjuvant treatment eligibility, it is known that a subset of patients with early stage disease experience shorter survival times than others with the same clinicopathological characteristics. Improved methods for identifying these individuals, at or near the time of their initial diagnosis, may support a decision to pursue an increased frequency of screening or use of adjuvant therapy options. The ultimate goal of this work is to provide a tool for generating personalized assessments of prognosis and adjuvant chemotherapy (ACT) response, particularly for patients with early stage disease, in order to reduce the rate of over and under treatment in NSCLC [2].

Subramanian and Simon recently compared 16 studies describing the development of prognostic gene expression signatures for NSCLC, published between 2002 and 2009 [3]. A standard set of assessment criteria was applied to each, including an evaluation of study design and statistical analysis methods, and whether the signature demonstrated an improvement over existing methods of prognosis. The study concluded that none of the expression signatures could demonstrate a significant improvement over a clinical formula based on the age and tumor size, and thus were not useful for clinical application [4].

Heterogeneity of response to ACT significantly confounds treatment for patients with NSCLC. As such, methods are needed to avoid unnecessary treatment in patients unlikely to respond, despite satisfying the current treatment guidelines for a given agent or combination of agents. Clinical trials conducted by multiple groups have shown a potential benefit of cisplatin-based ACT for individuals with completely resected tumors, ranging from a 4-15 % survival benefit [5,6]. Unfortunately no significant benefit for patients with stage I NSCLC has been observed to date, and as such the standard of care for these patients is surgery and observation [7].

In a uniquely designed, randomized controlled clinical trial, Zhu et al. identified 15 genes which stratify patients into groups distinguished by a significant difference in both outcome and adjuvant cisplatin/vinorelbine benefit [8]. While the prognostic ability of the 15-gene algorithm was independently validated using a previously published series of NSCLC patients, only internal cross-validation results were presented to verify the signatures ability to predict response to ACT. While a correctly conducted cross-validation approach may give an initial unbiased estimate of classifier accuracy, predictive algorithm validation using at least one external, independent patient series is recommended [3,9]. Analysis of data from patients not used in the gene selection and/or algorithm training allows assessment of the impact of ‘real-world’ technical and biological variation on the performance of a novel multi-gene assay.

Therefore, the goal of this study was to develop and validate complimentary algorithms for (i) stratifying stage I-II NSCLC patients into categories with significant differences in disease-specific survival (DSS) and (ii) stratifying stage I-III patients on the basis of cisplatin-based ACT-benefit, defined as treatment-related change in DSS. The analytical guidelines proposed by Subramanian and Simon were followed closely throughout, in order to maximize the clinical relevance of the novel algorithms developed. Finally, it was hypothesized that prognosis and sensitivity to ACT agents may represent independent characteristics of NSCLC. If this were to be the case, patients with good or bad prognosis may be equally likely to possess the molecular characteristics required for ACT-induced tumor cell death, requiring separate but complimentary algorithms for the optimal prediction of prognosis and treatment response.

## Methods

### Compilation of a genomic database for gene selection & algorithm training

**Figure 1:**
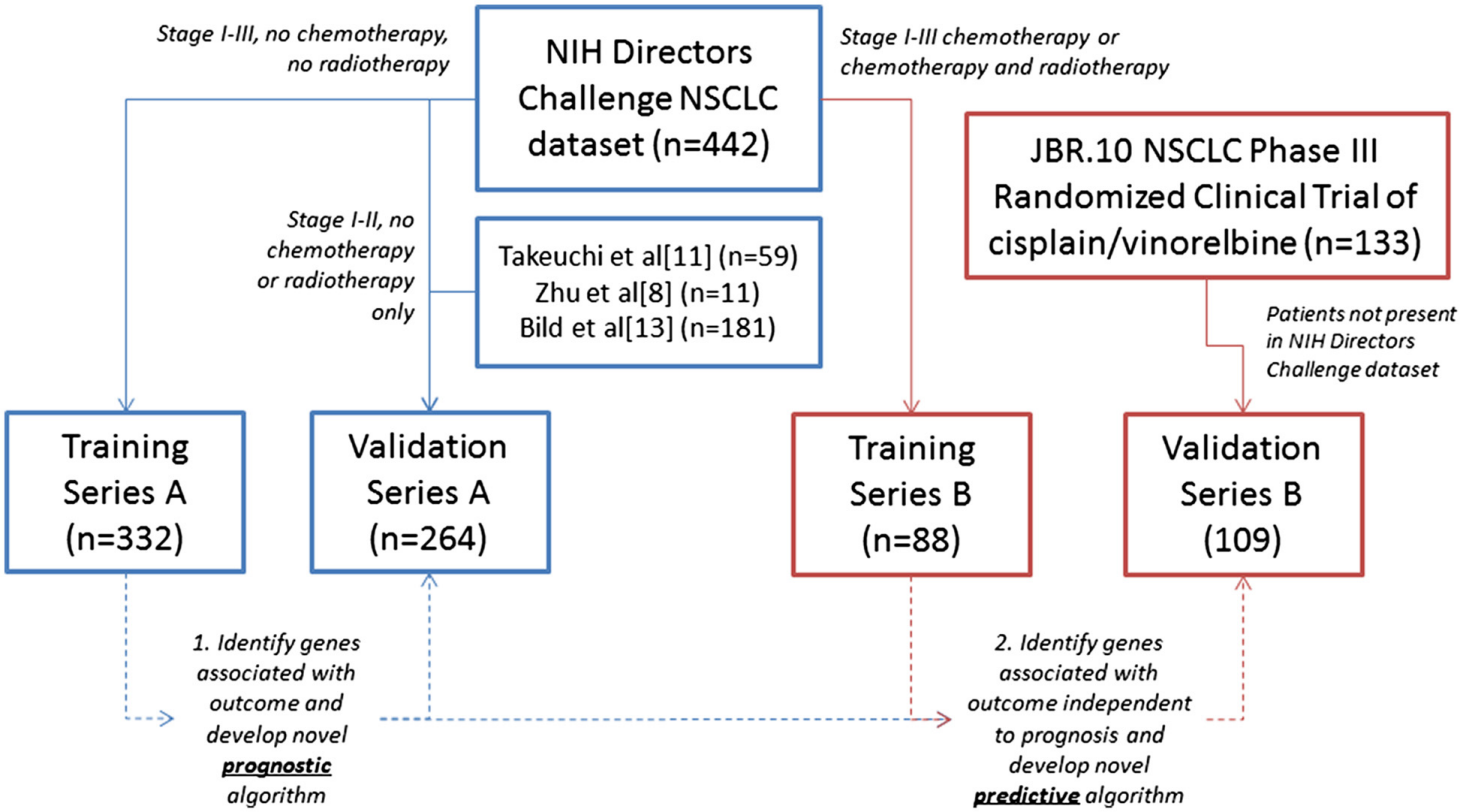
**Schematic diagram of datasets used to form training and validation series used in this study.** Data from treatment-naïve adenocarcinoma patients enrolled in the NIH Director's Challenge Consortium for the Molecular Classification of Lung Adenocarcinoma were first used to develop a prognostic signature able to predict DSS, independent to clinical factors such as age and clinical stage [10]. This signature was validated on the independent adenocarcinoma series listed and then used to identify a new set of genes from ACT-treated patients that were associated with outcome, independent to prognosis. The second algorithm (ACT-response) was validated on data from Zhu et al. [8].

Genomic and clinical data from 420 patients who were originally part of The Director’s Challenge Consortium for Molecular Classification of Lung Adenocarcinoma (DCC) series (total N = 442) were used to identify two sets of genes associated with (a) disease-specific survival (DSS) and (b) response to ACT [10]. Patient details for the training and validation series used in both analyses are summarized in Table 1 and represented schematically in Figure 1. As reported in the original studies, consent was obtained for all subjects using protocols approved by each institution’s Institutional Review Board.

**Table 1:** Clinicopathological characteristics of the NSCLC patients used in this study (n/a = data unavailable)

**Variable**	**Prognostic signature**		**Chemotherapy-response signature**	
	**Training Series A (n = 332)**	**Validation Series A (n = 264)**	**Training Series B (n = 88)**	**Validation Series B (n = 109)**
Age: Median (SD)	65 (10)	64 (10)	62 (10)	69 (9)
Gender: Female, Male	155 (47 %), 177 (53 %)	141 (53 %), 123 (47 %)	51 (58 %), 39 (42 %)	30 (28 %), 79 (72 %)
AJCC Stage:	I: 229 (69 %), II: 61 (18 %), III: 42 (13 %)	I: 201 (75 %), II: 63 (25 %)	I: 39 (44 %), II: 27 (31 %), III: 21 (24 %), IV: 1 (1 %)	I: 57 (52 %), II: 52 (48 %)
Stage I: A/B	108, 121	93, 97	5, 34	n/a
Stage II: A/B	50, 11	13, 44	25, 3	n/a
Grade: 1/2/3/na	48 (14 %), 163 (49 %), 117 (35 %), 4 (<1 %)	58 (22 %), 94 (35 %), 62 (23 %), 53 (20 %)	10 (11 %), 40 (45 %), 36 (41 %), 2 (2 %)	n/a
Histological subtype	Adenocarcinoma: 332 (100 %)	Adenocarcinoma: 264 (100 %)	Adenocarcinoma: 88 (100 %)	Adenocarcinoma: 47 (43 %), Large cell: 10 (9 %), Squamous: 52 (48 %)
Smoking history	Never: 33 (10 %) Former: 181 (55 %), Current: 25 (8 %), Unknown: 90 (27 %)	Never: 43 (16 %), Former/current: 170 (64 %), Unknown: 54 (20 %)	Never: 14 (16 %) Former: 65 (74 %) Current: 7 (8 %) Unknown: 2 (2 %)	n/a
Radiotherapy	0	13 (5 %)	45 (51 %)	0
Chemotherapy	0	0	88 (100 %)	49 (45 %)
Original publication(s):	Sheddon et al. [10]	Sheddon et al. [10] Takeuchi et al. [11] Zhu et al. [8] Bild et al. [12] Bhattacharjee et al. [13]	Sheddon et al. [10]	Zhu et al. [8]
Genomic platform:	Affymetrix U133A	Agilent custom array: 59 (22 %), Affymetrix U95A: 140 (53 %), U133A/Plus 2.0: 65 (25 %)	Affymetrix U133A	Affymetrix U133A
NCBI GEO ID(s) or data source	NIH.gov^1^	GSE11969, GSE14814, GSE3141, http://broad.org/MRP/lung and NIH.gov^1^	NIH.gov^1^	GSE14814
Disease specific death within 5 years	122 (37 %)	97 (37 %)	47 (53 %)	34 (31 %)

^1^https://array.nci.nih.gov/caarray/project/details.action?project.experiment.publicIdentifier=jacob-00182.

A prognostic algorithm training series was created using genomic and clinical data from 332 DCC stage IA-IIIB patients who did not receive ACT or radiotherapy (Training Series A). This training series included patients with more advanced NSCLC (Stage IIIB) in order to capture a broad range of progression-related genomic information. A separate series of non-ACT treated 264 stage IA-IIB adenocarcinoma genomic profiles (Validation Series A) was compiled from five published studies in order to validate the prognostic signature on an independent series of patients [8,10-13]. Only patients diagnosed with stage IA-IIB NSCLC (and not used in Training Series A) were selected in order to reflect the intended use of the prognostic algorithm.

To create a multi-gene signature able to predict response to platinum-based ACT, a second training series was formed using those patients from the NIH Directors Challenge study who were treated with ACT and with data available for age at diagnosis, smoking status, tumor stage and outcome (Training Series B; n = 88, Figure 1). Sample annotation records indicate that cisplatin-based ACT was used for 24/88 patients, and although no specific agent information was available for the other individuals, presumably a standard-of-care platinum-based therapy was also used. To validate the predictive mult-gene signature identified from analysis of Training Series B, an independent validation series was used. This was comprised of pre-treatment genomic profiles from 109 patients with stage I-II disease who were enrolled in a randomized controlled trial of adjuvant cisplatin/vinorelbine (n = 49) *vs.* observation alone (n = 60) (Validation Series B) [8]. This previously published clinical trial series originally included genomic profiles from 133 patients; however 24 ACT-treated individuals were also enrolled in the NIH Directors Challenge study and were therefore included in Training Series B, which was used to identify the predictive signature. To avoid the possibility of bias by training and testing on data from the same individuals, these 24 patients were not included in the Validation Series B.

After stratifying patients in Validation Series B into predicted responders and non-responders, differences in DSS between those patients receiving ACT or OBS were compared using Kaplan Meier analysis and multivariate cox proportional hazards analysis.

### Development and validation of a gene expression signature to predict prognosis in patients with stage I-II lung adenocarcinoma

Genomic, clinical and outcome data from Training Series A (n = 332) were analyzed to identify genes with individual prognostic significance, using a method developed by Bair and Tibshirani [14] and used previously to develop prognostic algorithms for breast and colon cancer [15,16]. Briefly, genes were selected for inclusion in the prognostic signature if they were associated with outcome in Cox regression models at P < 0.001, independent to age at diagnosis, smoking history, gender, histological grade and AJCC stage [17,18]. Using 10-fold cross-validation, genes found to be significantly associated with outcome in two or more rounds of cross-validation were recorded and then used to train a principal component algorithm (PCA) [19]. At the completion of the gene selection process and prior to training of the final algorithm, expression data for the prognostic gene set were stabilized by conversion to percent-rank values, as previously described [15].

**Figure 2:**
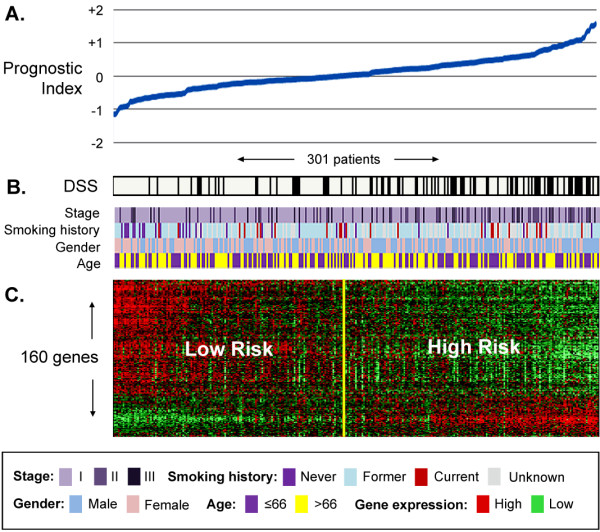
**Association between the 160-gene prognostic signature, clinical and survival information in 301 untreated lung adenocarcinoma patients from Training Series A patients with at least 12 months follow-up).** (**A**) Prognostic indexes range from −2 to +2 and are associated with an increase in DSS events, as indicated with a black line at (**B**). (**C**) Median-centered 160-gene expression profile used to compute the prognostic index (red = relative high expression, green = relative low expression). Each gene in the signature was chosen based on its statistically significant association with outcome, independent to age, stage, grade, gender and smoking history.

The output of the prognostic algorithm is a patient-specific ‘prognostic index’, ranging from −2.0 to 2.0 and continuously associated with risk of death from NSCLC, as reflected in Figure 2. To assign a patient to either a high or low risk group, their prognostic index is compared to a predetermined classification threshold. For this study, the threshold was set at the 60^th^ percentile of prognostic indexes observed for Training Series A. The prognostic algorithm was independently evaluated by applying it to data from Validation Series A, which comprised of 264 stage I-II adenocarcinoma patients who were not used in the gene selection or algorithm training process.

### Development and validation of a comparator ‘clinical algorithm’ for predicting prognosis in patients with early-stage NSCLC

A key criterion for evaluating NSCLC prognostic gene expression assays is the ability to improve over current ‘clinical’ methods of identifying patients with stage 1 disease at high risk of DSS (*i.e.* poor prognosis). To compare the novel prognostic signature developed herein with a clinical assessment of prognosis, the approach described in Subramanian & Simon *i.e.* a regression equation based on tumor size (≤3 cm or >3 cm) and age at diagnosis to predict prognosis was developed [3]. This algorithm was trained on age and tumor size data using the stage I patients from Training Series A. Cross-validation results were compared to those reported by Subramanian & Simon to ensure equivalency. Finally, accuracy of the clinical algorithm was evaluated by applying it to stage I patients from Validation Series A.

### Development and validation of a second gene expression signature to predict adjuvant chemotherapy benefit

Data from Training Series B (n = 88) were analyzed to select genes associated with outcome (DSS) in the clinical setting of ACT-treatment. To identify genes involved in ACT-response and not simply prognosis, the covariates included in the Cox regression models were age, stage, gender, smoking history and prognosis risk group (P < 0.001), as determined by previously developed prognostic algorithm. A two principal component classifier was trained on the resulting gene selection, as described previously. The final classifier was applied to the Validation Series B, representing 109 patients enrolled in a randomized controlled trial of ACT *vs.* OBS [8].

The predictive index generated by this secondary algorithm classifies patients as either ‘ACT-responders’ or ‘ACT-non-responders’, depending on whether the index is above or below the predetermined classification threshold (median index of Training Series B). Within each prediction category, Kaplan Meier analysis with log rank testing and Cox proportional hazards analysis was used to compare the rates of DSS for ACT and OBS treatment arms.

### Data processing and probe selection

For Affymetrix datasets, raw CEL files were downloaded and processed using the MAS5 algorithm. Datasets were median-centered within each microarray type. NCBI UniGene build #230 was used to assign gene annotation data to microarray features and match data between platforms. Probeset redundancy (where present) was reduced by identifying the probe with the highest mean intensity across all samples. Data stabilization was performed using the percentrank method (‘PERCENTRANK’ in Microsoft Excel 2010, ‘ecdf’ in R) as previously described [15].

### Statistical analysis and software

Gene expression data were analyzed using R 2.12 (http://www.r-project.org), Bioconductor [20] and BRB ArrayTools 4.2 [17]. Statistical analyses were performed using MedCalc 12.1.1 (MedCalc Software, Mariakerke, Belgium) and Microsoft Excel 2010 (Microsoft, Redmond, WA). Kaplan Meier analysis with log rank testing and multivariate Cox Proportional Hazards analysis was used to analyze the significance of prognostic and ACT-response risk group stratifications, with survival data censored at 60 months for prognosis prediction and 36 months for treatment response prediction. Receiver Operator Curve (ROC) analysis was used to compare on the gene expression and clinical-variable prognostic algorithms.

## Results

### Identifying genes associated with DSS and prognostic algorithm training

A cross-validated multivariate cox regression based method of gene selection was applied to 332 untreated stage I-III NSCLC whole genomic profiles (Training Series A) and a set of 160 unique genes was identified (Additional file 1 Table S5). Each gene was significantly associated with DSS independent of age at diagnosis, disease stage and gender at or below P < 0.001 (full list of genes provided in Additional file 1). Normalized log intensity values were stabilized by conversion to percent-rank values (range 0.000 to 1.000) and used to train a principal component algorithm able to classify a new patient as either high or low probability of death from lung cancer. The relationship between the 160-gene expression profile, corresponding prognostic index and the DSS of each patient in Training Series A is visualized in in Figure 2. A multivariate analysis of the cross-validated Training Series A risk group predictions is shown in Additional file 1 Table S1.

### Gene ontology characterization of the 160-gene prognostic signature

Functional characterization of the 160 prognostic genes was performed using the Database for Annotation, Visualization and Integrated Discovery (DAVID) v6.7 [21]. This system performed clustering of gene annotation terms associated with the 160-gene signature and showed an over-representation of genes involved in regulation of metabolic processes (enrichment score: 4.31), cellular organization (1.52), cell cycle control (1.25) and apoptosis (1.15).

Genes implicated in the MAPK signaling pathway (*i.e. CDC42MKNK1MAPKAPK2* and *TRADD*) were also over-represented in the gene set, compared to random selection (P = 0.034). Activation of the MAPK signaling pathway is linked to the oncogenic factor EAPII (*TDP2*) and the development of lung cancer [22].

Only one gene, TRIM14, was found to be in common between the 160-gene prognosis signature and the 15-gene signature of Zhu et al. [8]. This is a poorly-characterized gene that encodes for a protein which localizes to cytoplasmic bodies [RefSeq, Mar 2010].

### Independent validation of the 160-gene prognostic signature

To determine the ability of the prognostic signature to predict risk of DSS in patients not involved in the gene selection or training process, it was applied to data from an independent series of 264 lung adenocarcinoma patients with stage I-II disease. These patients were compiled from five previously published studies, as described in Table 1 (Validation Series A). After annotating the gene expression data from each series of patients with UniGene annotations, it was determined that two of the microarray platforms present in the combined series did not contain features that corresponded to all 160 genes that were identified from Training Series A. The Affymetrix U95A microarray used by Bhattacharjee et al. [13] contained 132/160 (83 %) of the genes while the custom Agilent format used by Takeuchi et al. [11] contained 135/160 (84 %). Rather than impute missing values using a k-NN method for example, it was decided to compute the prognostic score using the signature genes that were available. In this way the validation performed reflects conditions that may occur in real world use of such a multi-gene assay, in which variations in specimen preparation and microarray fabrication may lead to one or more missing data points per signature.

**Figure 3:**
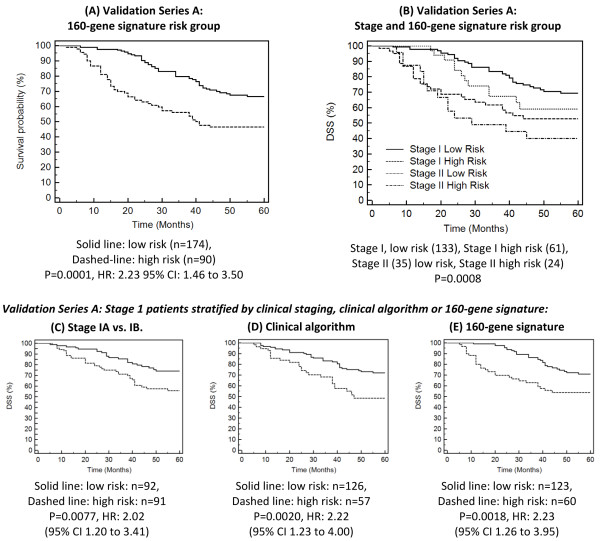
**Kaplan Meier analysis of Validation Series A patients, stratified by gene expression risk group (A) and clinical stage (B).** Kaplan Meier analysis was also performed on Stage IA patients from Validation Series A Stage stratified by AJCC stage (**C**), a clinical algorithm based on tumor size and age (**D**) and the 160-gene signature (**C**) for comparison purposes. The gene expression signature is able to more accurately identify stage I patients at risk of death within the first 24 months following diagnosis compared with clinical stage or combined clinical age + tumor size algorithm.

Of the 264 Stage I-II NSCLC patients in Validation Series A, 174 (66 %) patients were assigned to the low risk (good prognosis) category and 90 (34 %) to the high-risk (poor prognosis) category. Kaplan Meier analysis (Figure 3a) showed the difference in DSS between risk groups to be highly significant (P = 0.0001, HR: 2.23 95 % CI: 1.46 to 3.50). Furthermore, when adjusted for other prognostic factors such as age, gender, AJCC Stage, radiotherapy status and also microarray-type, the 160-gene signature was the strongest and most significant predictor of outcome (P < 0.0001, HR: 2.80, 95 % CI: 1.83 to 4.28, see Additional file 1 Table S1 for more details).

CPH analysis was also carried out on stage-based subsets of Validation Series A, in order to further characterize the prognostic significance of the 160-gene algorithm. Results shown in Table 2 indicate that when adjusted for covariates, the 160-gene signature is able to significantly stratify patient with IA, IB and IIA disease, in addition to stage I and II combined.

**Table 2:** Analysis of the independent Validation Series A risk group predictions generated using the 160-gene prognostic signature

**Stage**	**No. Patients**	**Kaplan Meier Analysis (160-gene signature assigned high/low risk categories)**		**Cox Proportional Hazards Regression (160-gene signature assigned high/low risk categories)**		**Receiver Operator Curve analysis (association with 5-year DSS)**	
		**Univariate P-value**	**Hazard Ratio (95 % CI)**	**Multivariate P-value**	**Hazard Ratio (95 % CI)**	**P-value**	**AUC (95 % CI)**
I & II	264	<0.0001	2.26 (1.46 to 3.50)	<0.0001	2.80 (1.83 to 4.28)	0.0004	0.66 (0.59 to 0.71)
I	201	0.0008	2.23 (1.30 to 3.84)	<0.0001	3.00 (1.78 to 5.08)	0.0002	0.68 (0.61 to 0.75)
IA	93	0.18	1.76 (0.70 to 4.47)	0.045	2.65 (1.029 to 6.83)	0.019	0.69 (0.59 to 0.78)
IB	97	0.0008	2.79 (1.38 to 5.64)	<0.0001	5.44 (2.48 to 11.97)	<0.0001	0.75 (0.65 to 0.83)
II	63	0.048	2.00 (0.98 to 4.14)	0.042	2.20 (1.034 to 4.69)	0.56	0.56 (0.42 to 0.70)
IIA	13	0.0097	5.57(1.59 to 19.59)	0.048	28.21 (1.048 to 759.30)	1.0	0.50 (0.17 to 0.83)
IIB	44	0.42	1.47 (0.56 to 3.83)	0.48	1.44 (0.52 to 4.027)	0.57	0.57 (0.40 to 0.58)

Cox Proportional Regression analysis included stage*, microarray type, gender, treatment and age. *where possible, eg.stage IA *vs.* IB were in the analysis of all Stage I patients.

Receiver Operator Curve (ROC) analysis also confirmed the prognostic index to be a continuous predictor of outcome (Area Under the Curve (AUC) for all Stage I-II Validation Series B patients = 0.66, =0.0004, 95 % CI: 0.59 to 0.71), excluding patients alive but with less than 12-months follow-up or death from lung cancer after 36 months. Using a 24 month cut-off for death from lung cancer, the AUC increases to 0.74 (P < 0.0001, 95 % CI: 0.67 to 0.80), suggesting increased accuracy at identifying early-stage patients at short term risk of cancer-related death.

### Comparison of gene expression *vs.* clinical prognostic algorithms

Utility of new prognostic methods for NSCLC is influenced by their extent of improvement upon currently accepted approaches. To compare the 160 gene signature against prognosis based on clinical assessments, an algorithm based on age at diagnosis and tumor size (≤3 cm or >3 cm) was developed on the 195 Stage I patients from Training Series A, using the method described by Subramanian and Simon [3]. The clinical algorithm stratified Stage I patients from Validation Series A (Figure 3D) into groups with statistically significant difference in DSS (P = 0.004, HR: 2.65 95 % CI 1.40 to 1.99).

Comparing Kaplan Meier curves for gene expression and clinical algorithms (Figure 3C-E) illustrates an important difference between DSS prediction methods; the 160-gene signature is superior to either staging (IA *vs.* IB), or the clinical algorithm at identifying stage I patients at risk of death within 24 months. Of the 5 Validation Series A patients who were diagnosed with stage IA cancer and died within 24 months of diagnosis, all 5 were correctly predicted to be high-risk by 160-gene signature. When the clinical algorithm was applied to the same patients, only 2 of the 5 were classified as high-risk. Conversely, none of the stage IA patients predicted by the gene-signature to be low-risk (n = 65) died of their disease during the same 24-month time period (Figure 3E). This ability of the gene signature to identify early-stage individuals at high risk of death within a relatively short time frame may represent an opportunity for clinical intervention, such as the use of adjuvant chemotherapy.

ROC analysis was also performed to compare the genomic and clinical prognostic algorithms on stage I patients. For DSS within 5 years following diagnosis, both methods resulted in a similar AUC (Genomic: 0.66 Clinical: 0.64, P-value for difference: 0.75). When considering the ability to predict DSS within two years the difference was more apparent (Genomic: 0.74 Clinical: 0.61, P-value for difference: 0.083). Finally, Cox proportional hazards analysis of stage I patients was performed, evaluating gender and both genomic and clinical algorithms simultaneously. This revealed the gene signature to be the strongest and most significant predictor of outcome (genomic algorithm HR: 2.70 95 % CI: 1.55 to 4.65 P = 0.0005, clinical algorithm HR: 2.20 95 % CI: 1.27 to 3.68, P = 0.0047).

### Identifying genes related to ACT-response and predictive algorithm training

To discover genes with patterns of expression correlated with future response to ACT, a multivariate selection method was applied to data from 88 ACT-treated adenocarcinoma patients (Training Series B). By including each patient’s previously-determined 160-gene prognosis score in the gene selection algorithm, a cross-validated gene selection procedure identified 37 genes to be significantly associated with outcome, independent of age, stage, gender and prognosis (Additional file 1 Table S6). Kaplan Meier analysis of the (cross validated) Training Series B risk group assignments made during the training process revealed a significant difference in DSS between high and low risk groups (P = 0.0021, HR: 2.48, 95 % CI: 1.40 to 4.42). As all patients in Training Series B received ACT and the genes selected were related to outcome independent of prognosis, it was hypothesized that the difference in DSS between risk groups reflected the benefit of ACT in these individuals. This hypothesis was tested by applying the 37-gene signature to Validation Series B, comprised of individuals enrolled in a randomized clinical trial of ACT (cisplatin/vinorelbine) *vs.* OBS.

### Functional characterization of the 37-gene ACT response signature and overlap with the 160-gene prognosis signature

Analysis of gene function using DAVID showed the 37-gene signature contained genes with functions previously linked to vinorelbine and/or cisplatin efficacy, including lipid metabolism (eg. *LARGEFA2H*, and *PCYT1B*) [23], membrane transport (eg. *SLC17A1COX4I1* and *SLC2A1*) [24], apoptosis and proliferation (eg. *CASP9DUSP22* and *TBX2*) [25] and purine binding (*DHX16* and *LYN*) [26]. An annotated list of the 37 genes, with Cox regression p-values, is provided in Additional file 1.

Despite starting with the same initial set of gene set, inspection of both prognostic and predictive algorithms revealed an overlap of only one gene; splA/ryanodine receptor domain and SOCS box containing 3 (*SPSB3)*. *SPSB3* has been shown to interact with MET and (based on protein structure) and is thought to be involved in ubiquitination and proteasomal degradation [27]. In this study, patients with good prognosis and predicted to respond to ACT had higher levels of *SPSB3* compared to those with poor prognosis and not likely to respond to ACT.

The 160 and 37-gene sets were also compared at the ontology and molecular pathway level using the Fatigo tool for identifying significant associations between groups of genes [28]. At the P < 0.05 significance level, no gene ontologies were significantly represented in both gene sets (levels 3–9 of ontology structure tested), nor were any of the KEGG or Biocarta molecular pathways. Fatigo results are provided in Additional file 2.

None of the 37 ACT-response genes overlapped with the 15 gene set described by Zhu et al. [8].

### Independent validation of the 37-gene predictive signature

To verify the ability of the novel 37-gene ACT-response signature, (identified from 88 ACT-treated adenocarcinoma patients; Training Series B), to stratify individuals into groups with different ACT response rates, an independent validation series was analyzed. The signature classified 70 patients from Validation Series B ACT-responders (64 %) and 39 as ACT-non-responders (36 %).

**Figure 4:**
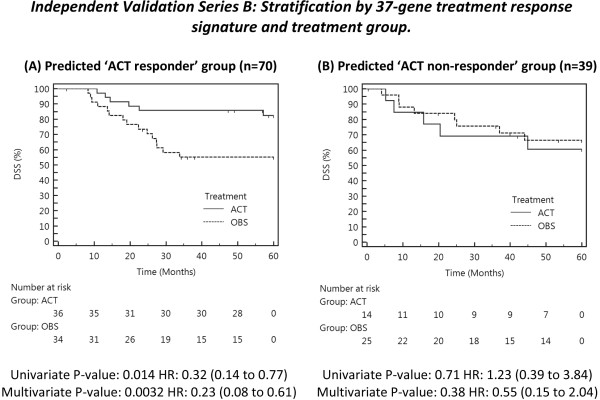
**Kaplan Meier analysis: 37-gene signature treatment response predictions for independent Validation Series B.** Patients in (**A**) Predicted ‘ACT-responder’ group exhibit significantly improved rate of DSS when treated with ACT compared to OBS alone. Patients in (**B**) Predicted ‘ACT non-responder’ group do not exhibit a significant difference in DSS between either treatment arm of the trial. Multivariate Cox Proportional Hazard analysis included age, gender, stage, NSCLC histological subtype and treatment (ACT or OBS).

Kaplan Meier analysis showed that the predicted ACT-responders experienced significantly greater DSS when treated with ACT, compared to predicted responders who received observation only (Figure 4). The difference was significant in both univariate (P = 0.014), and multivariate analysis (P = 0.0032), adjusted for age, gender, stage and histology. Inspection of hazard ratios showed that ACT-responders are at a 3.1-fold (unadjusted for clinical covariates) or 4.4-fold (adjusted) lower risk of death within 5-years, when treated with ACT. Full model results with 95 % CI’s are shown in Additional file 1.

For those individuals assigned to the ACT non-responders group (n = 39) no statistically significant difference in DSS was detected between ACT or OBS treatment arms (univariate P = 0.71, multivariate P = 0.38). Taken together, these findings confirm that the 37-gene signature can be used to select those individuals likely to benefit from cisplatin-based chemotherapy, who in this series represent 64 % of all stage IB-II patients.

Analysis of the stage I/II distribution between the predicted ACT response groups in Validation Series B confirms the findings of other groups that determining ACT-eligibility using clinical staging results in sub-optimal outcomes [5]. Thirty-eight of the 70 predicted ACT-responders were stage I (54 %), a group not usually considered eligible for ACT. Additionally, just over half of the Validation Series B patients predicted to be non-responders were diagnosed with stage II disease (n = 20). This implies that a quantifiable clinical benefit from ACT depends on the genomic profile of the tumor, rather than staging based on conventional assessment.

### Comparison of gene expression signatures in paired fresh-frozen and FFPE tissue

Both genomic signatures developed in this study were developed using data generated from fresh-frozen NSCLC tissue. For optimal clinical utility, a test based on FFPE tissue is preferred as collection of FFPE tissue is almost universal while frozen tissue is more difficult to transport and store. A preliminary comparison of the gene sets in paired samples of frozen and FFPE lung tissue was performed using previously published data from two lung tumors (NCBI GEO: GSE19249) [29]. Frozen and FFPE sections of each tumor were processed and hybridized to Affymetrix U133A GeneChips in triplicate.

Passing & Bablok regression was used to compare the prognostic and predictive indices of the frozen and FFPE specimens. No significant deviation from index linearity (P > 0.10) [30], nor change in prognosis/ACT-response group was observed (see Additional file 1). The linearity of the 160-gene prognostic index and 37-gene predictive index observed suggests that these tests may be informative using FFPE tissue for diagnostic gene expression analysis, although further validation is required.

## Discussion

New methods for predicting outcome (DSS) and response to chemotherapy are needed to improve management of patients with NSCLC. Two multi-gene algorithms have been developed to predict DSS and ACT benefit, using a previously published multi-center series of lung adenocarcinoma gene expression profiles. The 160 gene prognostic and 37 gene predictive gene sets identified by this study overlap by a single gene, SPSB3*,* but no functional ontologies or molecular pathways were found to be in common. SPSB3 is a largely uncharacterized gene not previously linked to NSCLC but in this study found to be associated with good prognosis and benefit from ACT. Several gene ontologies significantly represented by the 160 and 37 gene signatures have been linked to prognosis or ACT efficacy, including MAPK-pathway regulation, apoptosis, membrane transport and metabolic activity.

The prognostic and predictive signatures developed in this study differ from previously published methods in a number of key areas. Both were developed from NSCLC datasets comprised of a single histological subtype (NSCLC), using multivariate methods of gene selection on a large, well annotated training series originally designed to meet statistical sample-size requirements [31]. The methods developed in this study satisfy the Subramanian and Simon [3] criteria for evaluating NSCLC prognosis signatures (reproduced and annotated in Additional file 1). These include description of relevant patient characteristics (Table 1), no presentation of cross-validation statistics as the only performance metrics and the ability to apply the signature to other data for future comparisons and other non-clinical uses (http://www.ChipDX.com). Finally, the 160-gene prognosis signature has been shown to stratify stage IA, IB and II patients into groups with significant differences in RFS independent of clinical covariates.

The 160-gene prognostic signature was the single strongest predictor of outcome in patients with stage I disease when evaluated using multivariate Cox proportional hazards analysis (HR: 2.80, P = <0.0001). Furthermore, as shown by the comparison of ROC data and also in Figure 3C-E, the genomic method appears to be superior to other methods at identifying high-risk stage I patients, *i.e.* those at risk of death within 24 months. This may allow clinicians to recommend increased screening or the use of adjuvant chemotherapy in patients not otherwise considered eligible.

By evaluating the performance of the 160-gene prognosis signature on a multi-platform multi-center validation series, it has effectively been ‘stress-tested’ under conditions resembling real-world use. Despite the fact that 26 % of samples in Validation Series A were analyzed using microarrays without the complete 160-gene set, the classifier was shown to be the strongest predictor of outcome. Future validation studies using microarrays containing all 160 genes will help determine if the signature contains redundant information, or if the performance statistics generated herein are an underestimate the true prognostic significance of the algorithm.

The 37-gene ACT-response signature was developed using a novel approach to algorithm design - selecting genes associated with outcome in ACT treated patients, independent to a previously calculated prognosis score. By applying the response signature to an independent validation series of lung cancer patients who participated in a randomized clinical trial of cisplatin-based chemotherapy or observation only, the ability of the algorithm to identify those who would go on to receive a clinical benefit from ACT was demonstrated (HR: 0.23; P = 0.0032, adjusted for clinical covariates). To the contrary, those Validation Series B patients who were predicted to be non-responders, showed no difference in DSS between ACT and OBS arms of the trial (HR: 0.55; P = 0.38). None of the 37 genes overlapped with the 15-gene signature of Zhu et al. which was reported to have an ACT-benefit hazard ratio of 0.33 (P = 0.0005) for predicted responders and 3.67 (P = 0.013) for non-responders [8].

The use of ACT in stage I patients is currently controversial [5], however it is proposed that that the method described herein may allow clinicians to identify and treat only those individuals whose tumors have the molecular requirements of ACT efficacy. Prospectively planned trials and more extensive comparisons of data from frozen and FFPE tissues, additional NSCLC histologies and chemotherapeutic agents are necessary to further evaluate the clinical utility of the algorithms developed.

## Conclusions

This study describes novel genomic signatures able to significantly predict DSS and cisplatin-based ACT benefit for patients diagnosed stage I-II NSCLC. The signatures are comprised of biologically relevant genes and have been evaluated on genomic profile data obtained by multiple institutions using multiple microarray types, reflecting real-world usage. The distinct composition and lack of functional overlap of each signature supports the hypothesis that prognosis and response to ACT in NSCLC are factors influenced by unique molecular characteristics. In conclusion, robust multi-gene algorithms have been developed and validated on independent patient series, demonstrating the potential assist clinicians improve the management and treatment of patients diagnosed with NSCLC. Further work is required to confirm the findings reported herein and determine the applicability of these signatures for other lung cancer histologies and treatment modalities.

## Abbreviations

HR, Hazard ratio; NSCLC, Non-small cell lung cancer; DSS, Disease specific survival; ACT, Adjuvant chemotherapy treatment; OBS, Observation only (no chemotherapy).

## Competing interests

ChipDX is a self-funded start-up company based in New York and has applied for patent protection for the methods described in this manuscript.

## Authors’ contribution

RVL carried out all work on this paper.

## Authors’ information

Ryan Van Laar is the Director of Bioinformatics for Signal Genetics LLC, the parent company of ChipDX LLC.

## Pre-publication history

The pre-publication history for this paper can be accessed here:

http://www.biomedcentral.com/1755-8794/5/30/prepub
